# Comparison of the burden of digestive diseases between China and the United States from 1990 to 2019

**DOI:** 10.3389/fpubh.2024.1376406

**Published:** 2024-05-17

**Authors:** Jieyu Peng, Huan Xu, Shu Huang, Xiaomin Shi, Ping Wang, Qi Chen, Wei Zhang, Lei Shi, Yan Peng, Fangfang Yuan, Xiaowei Tang

**Affiliations:** ^1^Department of Gastroenterology, The Affiliated Hospital of Southwest Medical University, Luzhou, China; ^2^Nuclear Medicine and Molecular Imaging Key Laboratory of Sichuan Province, Luzhou, China; ^3^Department of Gastroenterology, Lianshui County People’s Hospital, Huaian, China; ^4^Department of Gastroenterology, Lianshui People’s Hospital of Kangda College Affiliated to Nanjing Medical University, Huaian, China; ^5^Department of Intensive Care Unit, The 3rd Xiangya Hospital, Central South University, Changsha, China

**Keywords:** digestive disease, Global Burden of Disease, average annual percentage change, China, the United States

## Abstract

**Introduction:**

China has experienced unprecedented transformations unseen in a century and is gradually progressing toward an emerging superpower. The epidemiological trends of digestive diseases in the United States (the US) have significant prescient effects on China.

**Methods:**

We extracted data on 18 digestive diseases from the Global Burden of Diseases 2019 Data Resource. Linear regression analysis conducted by the JoinPoint software assessed the average annual percentage change of the burden. We performed subgroup analyses based on sex and age group.

**Results:**

In 2019, there were 836.01 and 180.91 million new cases of digestive diseases in China and the US, causing 1558.01 and 339.54 thousand deaths. The age-standardized incidence rates of digestive diseases in China and the US were 58417.87/100,000 and 55018.65/100,000 respectively, resulting in age-standardized mortality rates of 81.52/100,000 and 60.88/100,000. The rates in China annually decreased by 2.149% for mortality and 2.611% for disability-adjusted life of year (DALY). The mortality and DALY rates of the US, respectively, had average annual percentage changes of −0.219 and −0.251. Enteric infections and cirrhosis and other chronic liver diseases accounted for the highest incidence and prevalence in both counties, respectively. The burden of multiple digestive diseases exhibited notable sex disparities. The middle-old persons had higher age-standardized prevalence rates.

**Conclusion:**

China bore a greater burden of digestive diseases, and the evolving patterns were more noticeable. Targeted interventions and urgent measures should be taken in both countries to address the specific burden of digestive diseases based on their different epidemic degree.

## Introduction

The extensive occurrence of digestive diseases and their potential to cause numerous fatalities and impairments have substantially burdened global public health. Previous studies have provided a comprehensive and systematic analysis of the worldwide burden of digestive diseases (1, 2). Investigations into digestive diseases, either on an individual or entire scale, in regions and countries at different development levels are also available (3–6). Additionally, globally, drug-induced digestive complications present additional challenges to disease management (7). Amid global changes in dietary patterns and lifestyles, appropriate dietary adjustments have been shown to effectively regulate disease severity and incidence, underscoring the pivotal role of dietary management in preventing gastrointestinal disorders (8, 9).

In the United States (the US), digestive diseases resulted in millions of healthcare visits and hundreds of thousands of deaths, with billions of dollars spent annually (10). A plethora of research has shown that a variety of digestive diseases remain a major challenge in China, thus necessitating the implementation of proactive disease prevention and control policies, additional epidemiological research and examination, drug creation, personnel development, and other health burden management strategies (11–14).

The US and China were the representatives of the largest developed and developing countries, respectively. The current state of health and epidemiological trends in the US could potentially shape the future of developing countries. In the US, the consequences of a well-established economic and social structure, a Western way of life, and a top-notch disease prevention and control system have led to the existing burden of digestive diseases. It could be seen as a reflection of the potential evolution of digestive diseases in the future of developing countries. In addition, China has gone through extraordinary changes that have not been witnessed in a century and is steadily advancing toward becoming the next major power. It is reasonable to compare the disease burden between these two countries. Earlier researches have documented the presence of individual or multiple digestive diseases in a single nation (11, 15) or compared two nations for specific digestive disease (16, 17). No research was conducted to compare the systematic burden of digestive diseases between China and the US, which was precisely what we were looking to investigate.

## Methods

### GBD review and data extraction

We obtained information regarding China and the US from the Global Burden of Disease Study 2019 Data Resources (GBD 2019, http://ghdx.healthdata.org/gbd-2019), which is sourced from the Global Health Data Exchange (GHDs), the most extensive collection of surveys, censuses, vital statistics, and other health-related data in the world. Health Metrics and Evaluation, an independent global health research center at the University of Washington, is responsible for the creation and support of GHDs. GBD 2019 quantifies health loss of 87 risk factors and 369 diseases and injuries across 204 countries and territories (18, 19). Statistical modellings of the cause of death ensemble model, spatiotemporal Gaussian process regression, and Bayesian meta-regression modelling tool were employed to estimate the input data from GBD 2019, utilizing a wide range of data sources including censuses, household surveys, civil registration, and vital statistics, disease registries, health service use, air pollution monitors, and other sources (19). GBD 2019 offers a platform for individuals with a keen interest in global health and demographics to swiftly access and exchange data, encompassing both datasets and relevant information.

The classification of diseases and injuries in GBD 2019 is based on four levels (19). This study analyzed a wide range of digestive diseases, including 17 level-3 causes: acute hepatitis; appendicitis; cirrhosis and other chronic liver diseases; colon and rectum cancer; eating disorders; esophageal cancer; gallbladder and biliary diseases; gallbladder and biliary tract cancer; inflammatory bowel disease; inguinal, femoral, and abdominal hernia; liver cancer; pancreatic cancer; pancreatitis; paralytic ileus and intestinal obstruction; stomach cancer; upper digestive system diseases; vascular intestinal disorders and one level-2 cause: enteric infections.

### Definitions

Four key estimations [incidence, mortality, prevalence, and disability-adjusted life of years (DALYs)] were analyzed in this study. The number of new cases during a given period in a specified population is known as incidence, and prevalence is the number of existing cases. Fatalities that take place in a population within a designated timeframe are counted as mortality. DALYs, defined as years of healthy life lost, are the total effect of quantity (premature mortality) and quality (disability) of life due to digestive diseases. Consequently, the computation of DALYs entails the summation of years lost due to premature death (YLLs) and years lived with disability (YLDs). YLLs are the multiplication of deaths and standard life expectancy at the age of death. YLDs are years lived with any health loss weighted for severity by disability weights, estimated by multiplying the prevalence with a distinct disability weight (20).

### Statistics

The age-standardized rate (per 100,000 persons) of the four aforementioned estimations was calculated based on the GBD world population age standard (21). The preceding research (22) has given an exhaustive description of the computation technique. The uncertainty intervals (UIs), derived from the 25th and 975th ordered 1,000 draw values of the posterior distribution (19), were presented for each of the estimations. The annual percentage change (APC) and average annual percentage change (AAPC) with a 95% certain interval (95% CI) from 1990 to 2019, calculated by linear regression analysis of the natural logarithm, were applied to analyze the overall trend and its turning points of the digestive disease burden. The calculation equations for APC and AAPC are outlined below (*i* stands for *i*-th year).


$${APC}_{i}=\left(\exp\left({\mathrm{beta}}_{i}\right)-1\right)*100$$

$$\mathrm{AAPC}=\left(\exp\left(\frac{\mathrm{sum}\left({\mathrm{beta}}_{i}*W_{i}\right)}{\mathrm{sum}\left(W\right)}\right)-1\right)*100$$

## Results

### Overall burden of digestive diseases in 2019

In 2019, there were 836.01 [95% UI: (749.70–931.33)] million new digestive cases in China, with an age-standardized incidence rate of 58417.87 (52149.69–65239.38) per 100,000 persons, resulting in a number of 1558.01 (1295.71–1837.47) thousand deaths and an age-standardized mortality rate of 81.52 (67.93–95.63) per 100,000 persons. The number of existing cases amounted to 612.90 (555.09–679.98) million, while the number of DALYs cases stood at 42.56 (34.75–51.45) million, resulting in age-standardized rates of 33813.21 (30703.83–37483.17) and 2188.77 (1790.35–2644.93) per 100,000 persons, respectively. In 2019, the US recorded 180.91 (166.90–196.73) million new cases, 339.54 (311.91–359.49) thousand deaths, 103.78 (93.29–115.96) million existing cases, and 8.59 (7.76–9.54) million DALYs, leading to age-standardized rates of 55018.65 (50511.52–60200.17), 60.88 (56.45–64.20), 25139.18 (22450.00–28094.35), and 1807.70 (1609.88–2042.67) per 100,000 persons, respectively (Tables 1, 2).

**Table 1:** Incidence, prevalence, deaths, and DALYs in 2019 for 18 individual digestive diseases in China.

	Incidence		Mortality		Prevalence		DALY	
	Number (millions)	ASR per 100,000 persons	Number (thousands)	ASR per 100,000 persons	Number (millions)	ASR per 100,000 persons	Number (millions)	ASR per 100,000 persons
Total digestive disease	836.01 (749.70–931.33)	58417.87 (52149.69–65239.38)	1558.01 (1295.71–1837.47)	81.52 (67.93–95.63)	612.90 (555.09–679.98)	33813.21 (30703.83–37483.17)	42.56 (34.75–51.45)	2188.77 (1790.35–2644.93)
Enteric infections	717.68 (647.65–794.71)	50626.74 (45322.15–56393.89)	6.68 (4.89–10.04)	0.55 (0.42–0.76)	10.04 (9.12–11.12)	724.04 (649.50–809.73)	1.41 (1.05–1.83)	110.98 (83.89–143.57)
Acute hepatitis	46.74 (40.46–53.40)	3768.44 (3358.34–4190.48)	3.73 (3.03–4.62)	0.20 (0.17–0.25)	4.50 (3.79–5.27)	344.83 (301.53–389.87)	0.21 (0.17–0.26)	13.85 (11.20–17.16)
Upper digestive system diseases	42.33 (37.49–47.79)	2342.99 (2082.70–2635.64)	58.69 (50.00–67.89)	3.44 (2.95–3.95)	91.52 (81.05–102.97)	5021.74 (4453.13–5636.20)	2.32 (1.85–3.03)	124.55 (99.03–162.23)
Gallbladder and biliary diseases	18.64 (15.53–22.54)	983.15 (827.97–1166.37)	16.02 (13.14–21.05)	1.04 (0.85–1.33)	66.41 (55.44–80.42)	3436.79 (2899.88–4138.41)	1.67 (1.15–2.36)	87.92 (61.06–122.86)
Appendicitis	2.49 (1.97–3.06)	177.03 (138.97–221.03)	1.57 (1.25–1.89)	0.10 (0.08–0.12)	0.10 (0.08–0.12)	6.74 (5.29–8.39)	0.07 (0.05–0.08)	4.31 (3.43–5.35)
Inguinal, femoral, and abdominal hernia	1.95 (1.51–2.42)	125.09 (100.53–149.98)	1.63 (1.28–2.08)	0.11 (0.08–0.14)	3.74 (3.02–4.54)	251.10 (204.15–306.17)	0.29 (0.20–0.39)	19.97 (13.88–27.93)
Paralytic ileus and intestinal obstruction	1.68 (1.62–1.75)	99.76 (96.56–103.11)	15.48 (12.67–18.45)	1.06 (0.87–1.23)	0.10 (0.09–0.10)	8.23 (7.75–8.70)	0.39 (0.33–0.46)	31.62 (26.84–36.37)
Eating disorders	1.57 (1.06–2.17)	143.28 (96.83–199.97)	0.04 (0.03–0.05)	0 (0–0)	1.50 (1.10–1.97)	112.62 (83.53–145.30)	0.32 (0.20–0.48)	24.54 (14.98–36.33)
Stomach cancer	0.61 (0.51–0.73)	30.64 (25.82–36.15)	421.54 (353.52–493.18)	21.72 (18.31–25.31)	1.44 (1.19–1.73)	70.06 (58.17–83.78)	9.82 (8.19–11.63)	481.15 (403.20–567.36)
Colon and rectum cancer	0.61 (0.52–0.71)	30.55 (26.37–35.50)	261.78 (224.40–303.32)	13.86 (11.92–16.01)	3.42 (2.93–3.99)	167.67 (143.72–194.53)	6.39 (5.46–7.41)	320.57 (275.40–370.70)
Pancreatitis	0.49 (0.42–0.58)	26.76 (22.76–31.25)	10.66 (8.20–12.81)	0.59 (0.46–0.70)	0.29 (0.26–0.34)	15.06 (13.13–17.03)	0.30 (0.24–0.36)	16.09 (12.73–19.36)
Cirrhosis and other chronic liver diseases	0.41 (0.31–0.51)	22.46 (17.56–27.59)	152.26 (128.22–179.74)	7.81 (6.62–9.16)	427.98 (395.52–465.24)	23561.25 (21807.83–25635.19)	4.34 (3.64–5.15)	217.77 (183.43–256.92)
Esophageal cancer	0.28 (0.21–0.33)	13.90 (10.70–16.52)	257.32 (202.78–309.03)	13.15 (10.27–15.68)	0.50 (0.38–0.60)	24.10 (18.24–28.75)	5.76 (4.58–7.00)	277.50 (221.69–335.87)
Liver cancer	0.21 (0.17–0.25)	10.46 (8.74–12.42)	187.70 (158.26–222.77)	9.41 (7.95–11.13)	0.29 (0.24–0.35)	14.38 (11.88–17.21)	5.33 (4.43–6.37)	264.31 (220.69–315.14)
Pancreatic cancer	0.11 (0.10–0.13)	5.78 (4.94–6.69)	117.37 (99.86–136.45)	5.99 (5.12–6.93)	0.09 (0.08–0.11)	4.46 (3.80–5.19)	2.81 (2.37–3.28)	136.57 (115.59–158.93)
Vascular intestinal disorders	0.11 (0.09–0.13)	5.84 (4.76–6.88)	6.41 (5.19–7.41)	0.37 (0.30–0.43)	0.01 (0.01–0.02)	0.69 (0.58–0.82)	0.12 (0.10–0.14)	6.24 (5.09–7.18)
Inflammatory bowel disease	0.05 (0.04–0.06)	3.01 (2.59–3.50)	4.68 (3.77–5.46)	0.30 (0.24–0.35)	0.91 (0.78–1.07)	47.06 (40.05–54.99)	0.23 (0.18–0.29)	13.10 (10.29–16.31)
Gallbladder and biliary tract cancer	0.04 (0.03–0.05)	2.01 (1.41–2.41)	34.46 (25.22–41.23)	1.82 (1.32–2.17)	0.05 (0.03–0.06)	2.40 (1.66–2.91)	0.76 (0.57–0.92)	37.71 (27.91–45.35)

DALY, disability-adjusted life years; ASR, age-standardized rate. The 95% uncertainty intervals were presented in parentheses.

**Table 2:** Incidence, prevalence, deaths, and DALYs in 2019 for 18 individual digestive diseases in the United States.

	Incidence		Mortality		Prevalence		DALY	
	Number (millions)	ASR per 100,000 persons	Number (thousands)	ASR per 100,000 persons	Number (millions)	ASR per 100,000 persons	Number (thousands)	ASR per 100,000 persons
Total digestive disease	180.91 (166.90–196.73)	55018.65 (50511.52–60200.17)	339.54 (311.91–359.49)	60.88 (56.45–64.20)	103.78 (93.29–115.96)	25139.18 (22450.00–28094.35)	8.59 (7.76–9.54)	1807.70 (1609.88–2042.67)
Enteric infections	152.82 (142.05–164.98)	47601.19 (44018.86–51749.70)	11.79 (10.32–12.69)	1.94 (1.72–2.08)	2.47 (2.35–2.59)	761.79 (717.92–807.83)	0.44 (0.36–0.54)	118.35 (92.17–149.78)
Upper digestive system diseases	16.71 (14.88–18.81)	4130.01 (3634.13–4682.61)	5.40 (4.80–5.82)	0.91 (0.83–0.98)	39.22 (34.96–44.06)	9547.90 (8425.77–10712.14)	0.48 (0.31–0.73)	111.63 (71.00–173.37)
Acute hepatitis	3.92 (3.59–4.24)	1426.33 (1282.78–1570.53)	0.13 (0.12–0.14)	0.03 (0.03–0.03)	0.32 (0.29–0.34)	113.91 (102.54–125.09)	0.01 (0.01–0.02)	3.90 (2.88–5.30)
Gallbladder and biliary diseases	3.65 (3.11–4.27)	853.84 (727.96–1000.81)	6.83 (5.70–7.90)	1.12 (0.95–1.30)	10.01 (8.97–11.31)	2349.21 (2086.94–2670.18)	0.31 (0.23–0.40)	67.41 (49.97–89.44)
Paralytic ileus and intestinal obstruction	1.08 (1.04–1.13)	240.60 (232.51–248.92)	12.04 (9.53–13.57)	2.04 (1.64–2.29)	0.05 (0.04–0.05)	11.40 (10.98–11.81)	0.20 (0.17–0.22)	46.49 (40.29–50.95)
Eating disorders	0.76 (0.52–1.06)	281.73 (191.12–396.07)	0.04 (0.04–0.05)	0.01 (0.01–0.01)	1.24 (0.92–1.58)	424.35 (316.44–540.37)	0.26 (0.17–0.39)	89.46 (56.85–131.63)
Appendicitis	0.51 (0.46–0.56)	160.91 (144.81–179.28)	0.65 (0.50–0.83)	0.12 (0.09–0.16)	0.02 (0.02–0.02)	6.13 (5.52–6.81)	0.02 (0.02–0.02)	5.32 (4.33–6.38)
Inguinal, femoral, and abdominal hernia	0.40 (0.32–0.49)	101.63 (82.81–121.82)	2.03 (1.72–2.22)	0.35 (0.30–0.38)	0.71 (0.59–0.84)	171.94 (144.24–201.95)	0.08 (0.06–0.10)	19.21 (15.17–23.88)
Vascular intestinal disorders	0.27 (0.24–0.30)	52.11 (46.79–57.08)	11.09 (9.89–11.83)	1.86 (1.68–1.98)	0.03 (0.02–0.03)	5.34 (4.88–5.85)	0.19 (0.18–0.20)	35.11 (32.80–36.78)
Pancreatitis	0.23 (0.21–0.25)	51.53 (47.37–56.19)	4.90 (4.50–5.39)	0.93 (0.87–1.02)	0.26 (0.24–0.29)	57.05 (52.54–61.38)	0.13 (0.12–0.15)	29.41 (27.28–32.57)
Colon and rectum cancer	0.23 (0.20–0.26)	41.86 (36.15–48.20)	84.03 (77.99–87.52)	14.77 (13.86–15.32)	1.38 (1.20–1.58)	259.23 (225.23–297.46)	1.76 (1.68–1.83)	338.87 (324.93–350.79)
Cirrhosis and other chronic liver diseases	0.09 (0.07–0.11)	26.33 (21.77–31.16)	67.29 (64.17–69.71)	13.18 (12.65–13.61)	47.08 (42.76–52.17)	11193.16 (10139.48–12393.78)	1.83 (1.77–1.88)	389.09 (377.01–400.40)
Inflammatory bowel disease	0.09 (0.08–0.09)	23.19 (21.18–25.62)	5.91 (4.62–6.46)	1.02 (0.80–1.11)	0.76 (0.71–0.81)	193.07 (179.65–206.87)	0.22 (0.18–0.26)	49.82 (40.25–60.19)
Pancreatic cancer	0.06 (0.05–0.07)	10.37 (8.94–11.96)	57.49 (53.67–60.25)	10.06 (9.43–10.52)	0.05 (0.04–0.06)	9.16 (7.89–10.58)	1.15 (1.09–1.19)	212.52 (202.97–220.25)
Stomach cancer	0.03 (0.03–0.04)	5.89 (5.10–6.87)	19.17 (17.82–20.05)	3.40 (3.19–3.54)	0.09 (0.07–0.10)	16.18 (13.93–18.94)	0.39 (0.37–0.40)	75.73 (72.84–78.30)
Liver cancer	0.03 (0.02–0.03)	5.23 (4.28–6.29)	23.81 (21.18–26.10)	4.33 (3.86–4.75)	0.04 (0.04–0.05)	8.48 (6.87–10.30)	0.55 (0.49–0.60)	107.18 (95.59–117.45)
Esophageal cancer	0.02 (0.02–0.03)	4.20 (3.54–4.98)	21.62 (20.55–22.52)	3.86 (3.69–4.02)	0.04 (0.03–0.05)	7.55 (6.34–8.96)	0.47 (0.45–0.49)	89.14 (85.88–92.25)
Gallbladder and biliary tract cancer	0.01 (0.01–0.01)	1.70 (1.43–2.08)	5.33 (4.78–6.45)	0.93 (0.83–1.12)	0.02 (0.02–0.02)	3.34 (2.83–4.06)	0.10 (0.10–0.12)	19.06 (17.66–22.95)

DALY, disability-adjusted life years; ASR, age-standardized rate. The 95% uncertainty intervals were presented in parentheses.

In 2019, the enteric infections accounted for the highest number of new digestive cases in both China [717.68 (647.65–794.71) million] and the US [152.82 (142.05–164.98) million], leading to age-standardized incidence rates of 50626.74 (45322.15–56393.89) and 47601.19 (44018.86–51749.70) per 100,000 persons, respectively. The prevalence of cirrhosis and other chronic liver diseases in both countries exhibited a similar pattern, with China recording 427.98 (395.52–465.24) million cases at an age-standardized rate of 23561.25 (21807.83–25635.19) per 100,000 persons, while the US recorded 47.08 (42.76–52.17) million cases at an age-standardized rate of 11193.16 (10139.48–12393.78) per 100,000 persons. For China, the most deaths [421.54 (353.52–493.18) thousand] and DALYs [9.82 (8.19–11.63) million] were observed in stomach cancer, which, respectively, caused age-standardized rates of 21.72 (18.31–25.31) and 481.15 (403.20–567.36) per 100,000 persons. For the US, the most significant death burden was observed in colon and rectum cancer with 84.03 (77.99–87.52) thousand cases and an age-standardized rate of 14.77 (13.86–15.32) per 100,000 persons, and the most considerable DALY burden was found in cirrhosis and other chronic liver diseases with 1.83 (1.77–1.88) million cases and an age-standardized rate of 389.09 (377.01–400.40) per 100,000 persons (Tables 1, 2).

### Trends of digestive disease burden from 1990 to 2019

**Figure 1:**
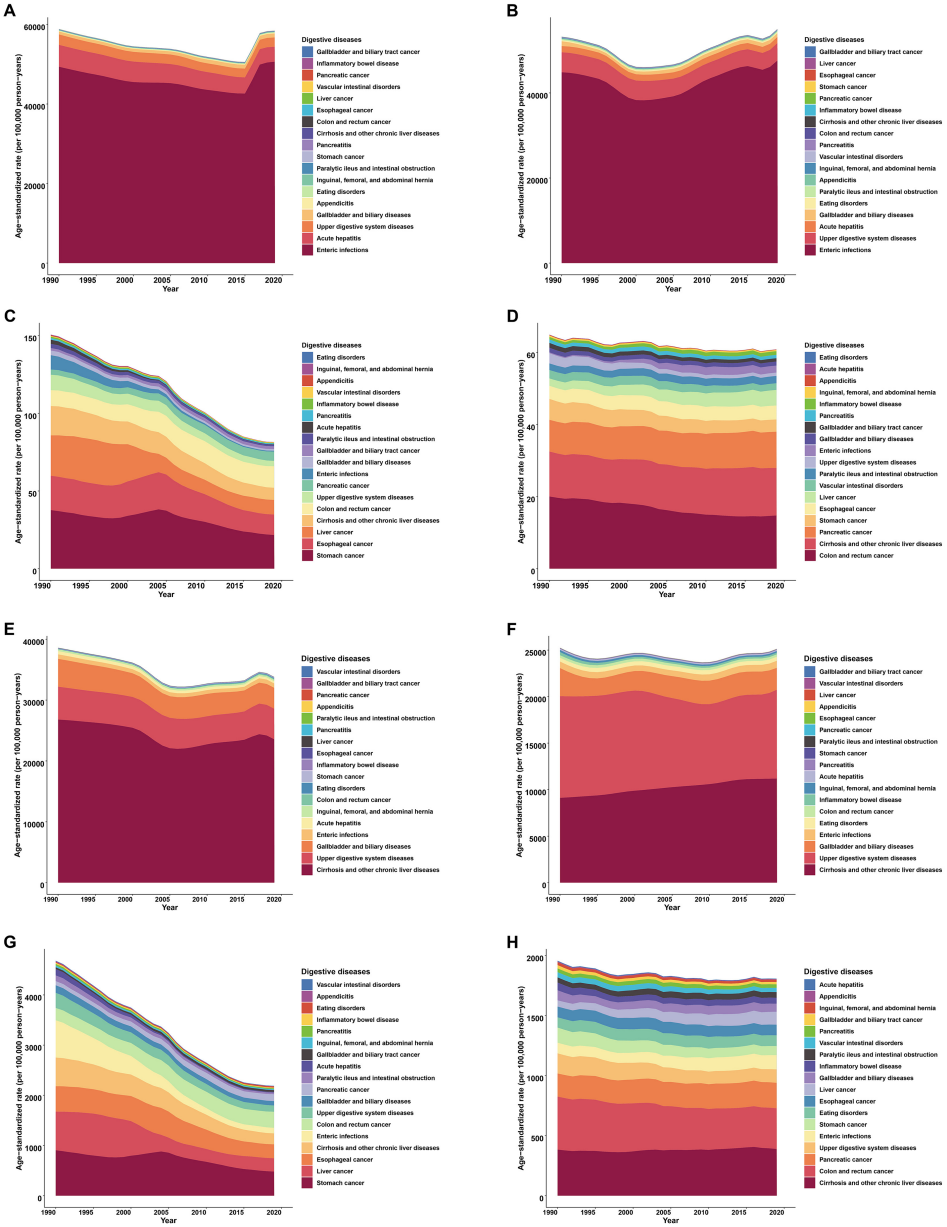
Contributions of individual digestive diseases to the overall age-standardized rates in China and the United States (the US), 1990–2019. **(A)** Incidence in China. **(B)** Incidence in the US. **(C)** Death in China. **(D)** Death in the US. **(E)** Prevalence in China. **(F)** Prevalence in the US. **(G)** DALY in China. **(H)** DALY in the US. DALY, disability-adjusted life year. The arrangement of the legends and stacked areas was done in increasing sequence based on the average values of the age-standardized rates obtained over a 30-year period.

Between 1990 and 2019, enteric infections, acute hepatitis, and upper digestive system diseases emerged as the primary culprits behind the emergence of new digestive diseases in both counties, with sequential differences in the higher burden of acute hepatitis in China. The three most prevalent diseases in both nations remain the same: cirrhosis and other chronic liver diseases, upper digestive system diseases, and gallbladder and biliary diseases. Despite this, there is a considerable disparity between the two countries in terms of the death and disability burden (Figure 1).

**Figure 2:**
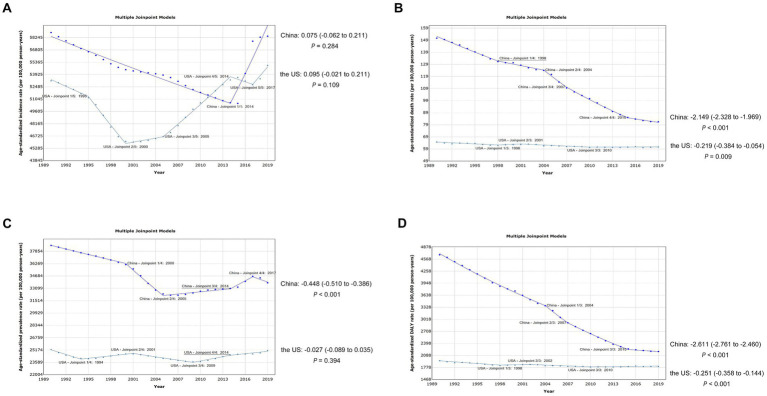
Trends of the overall burden of 18 digestive diseases in China and the United States, 1990–2019. **(A)** Trends of age-standardized incidence rates. **(B)** Trends of age-standardized death rates. **(C)** Trends of age-standardized prevalence rates. **(D)** Trends of age-standardized DALY rates. DALY, disability-adjusted life year.

Over the course of the last three decades, the age-standardized incidence rates of 18 digestive diseases in both China and the US have exhibited a pattern of initial increase followed by a subsequent decrease. In 2000, the US experienced a shift in its trend, which preceded China’s shift in 2004. Upon analyzing the prevalence, we discovered comparable patterns of burden alteration in both nations, with the exception of a marginal decline in the age-standardized rate in China [AAPC = −0.448 (−0.510 to −0.386), *p* < 0.001]. Significantly, there was a noticeable decrease in age-standardized mortality and DALY rates for both nations, particularly in China where the AAPC stood at −2.149 (−2.328 to −1.969, *p* < 0.001) for mortality and −2.611 (−2.761 to −2.460, *p* < 0.001) for DALY (Figure 2).

**Figure 3:**
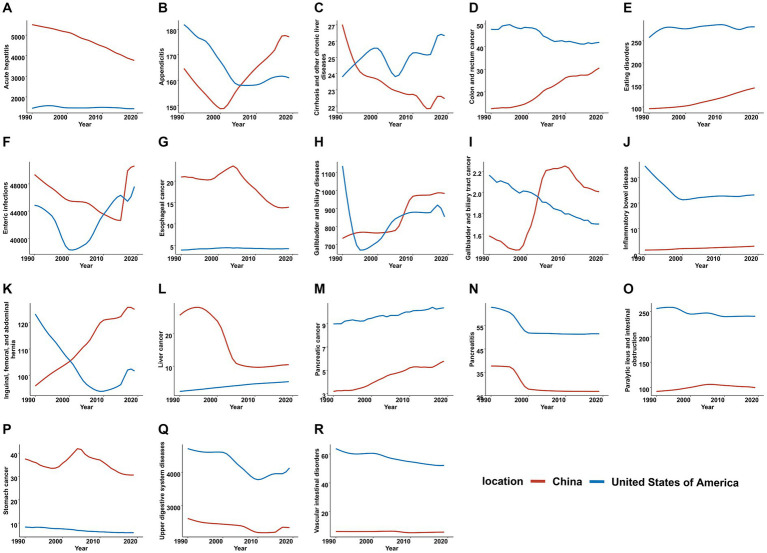
Trends of 18 individual digestive diseases in age-standardized incidence rates in China and the United States, 1990–2019. **(A)** Acute hepatitis. **(B)** Appendicitis. **(C)** Cirrhosis and other chronic liver diseases. **(D)** Colon and rectum cancer. **(E)** Eating disorders. **(F)** Enteric infections. **(G)** Esophageal cancer. **(H)** Gallbladder and biliary diseases. **(I)** Gallbladder and biliary tract cancer. **(J)** Inflammatory bowel disease. **(K)** Inguinal, femoral, and abdominal hernia. **(L)** Liver cancer. **(M)** Pancreatic cancer. **(N)** Pancreatitis. **(O)** Paralytic ileus and intestinal obstruction. **(P)** Stomach cancer. **(Q)** Upper digestive system diseases. **(R)** Vascular intestinal disorders.

The burden of individual digestive diseases fluctuated depending on the time period. From 1990 to 2019, colon and rectum cancer, enteric infections, inflammatory bowel disease, pancreatic cancer, pancreatitis, paralytic ileus and intestinal obstruction, upper digestive system diseases, and vascular intestinal disorders maintained a higher age-standardized incidence rate in the US, while China had a higher rate of acute hepatitis, esophageal cancer, liver cancer, and stomach cancer (Figure 3). The age-standardized prevalence rates also exhibited similar circumstances, while the convergence of the rate curves in the two nations progressively diminished (Supplementary Figure S1). The majority of digestive diseases in China showed a positive decrease in age-standardized mortality and DALY rates, with enteric infections (AAPC = −9.32, *p* < 0.001) and acute hepatitis (AAPC = −7.35, *p* < 0.001) respectively showing the most significant changes, except for colon and rectum cancer, eating disorders, gallbladder and biliary tract cancer, and pancreatic cancer, which saw an increase in both age-standardized mortality and DALY rates (Supplementary Figures S2, S3 and Supplementary Table S1). For the US, the rising age-standardized mortality rate in enteric infections (AAPC = 7.17, *p* < 0.001) marked a significant shift (Supplementary Figure S2 and Supplementary Table S2).

### Digestive disease burden stratified by sex and age

**Figure 4:**
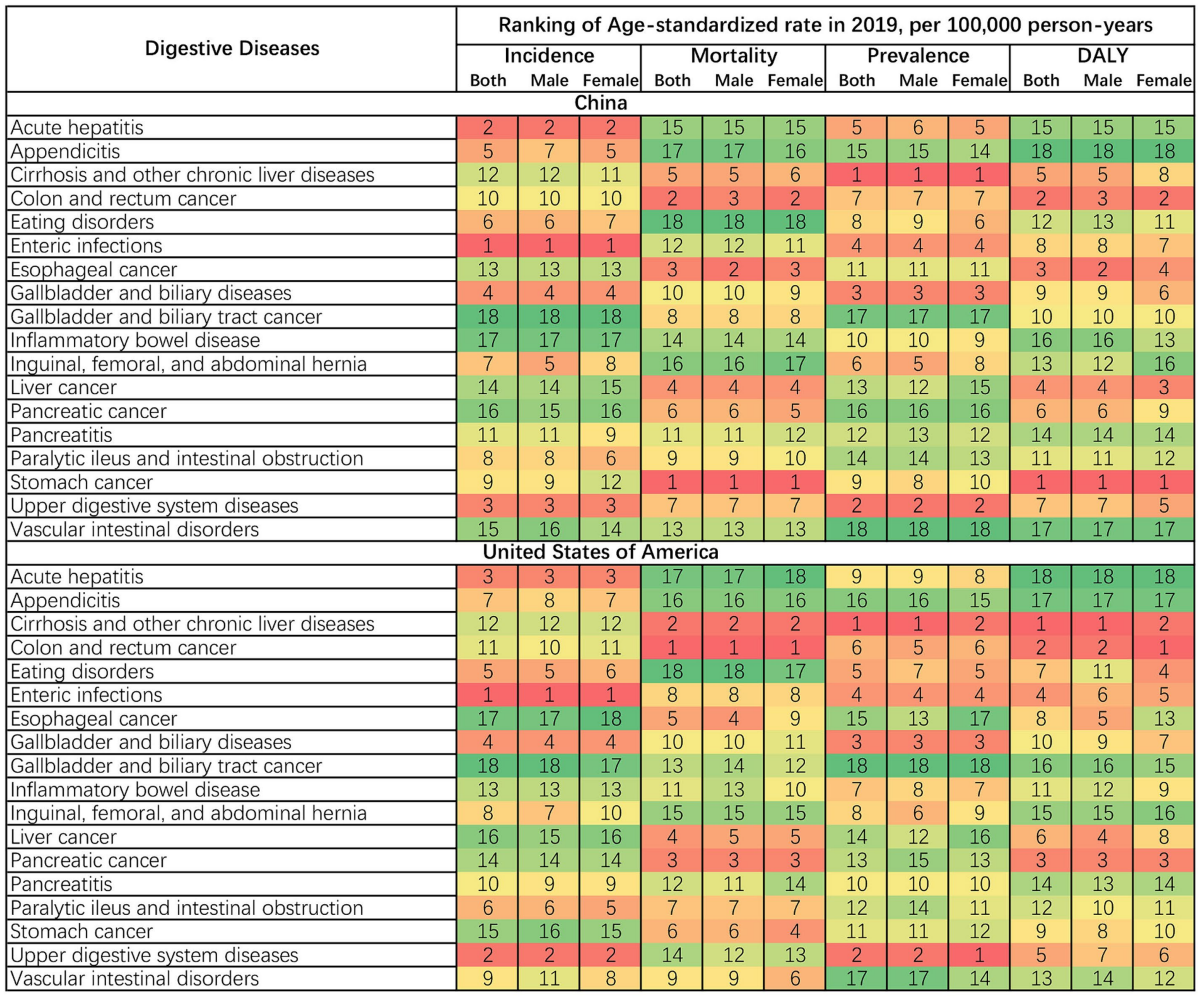
Ranking of age-standardized rates of incidence, mortality, prevalence, and DALY in digestive diseases by sex in 2019. DALY, disability-adjusted life year.

Although the age-standardized rates of 18 digestive diseases among different sexes were similar, there were still some differences. In China, esophageal cancer had a higher rank in age-standardized mortality and DALY rate among males, while colon and rectum cancer had a lower rank. A higher rank of age-standardized prevalence rate in upper digestive system disease and DALY rate in colon and rectum cancer among females was observed in the US. Additionally, in the US, females exhibited lower ranks of age-standardized prevalence and DALY rates in cirrhosis and other chronic liver diseases when compared with males (Figure 4).

**Figure 5:**
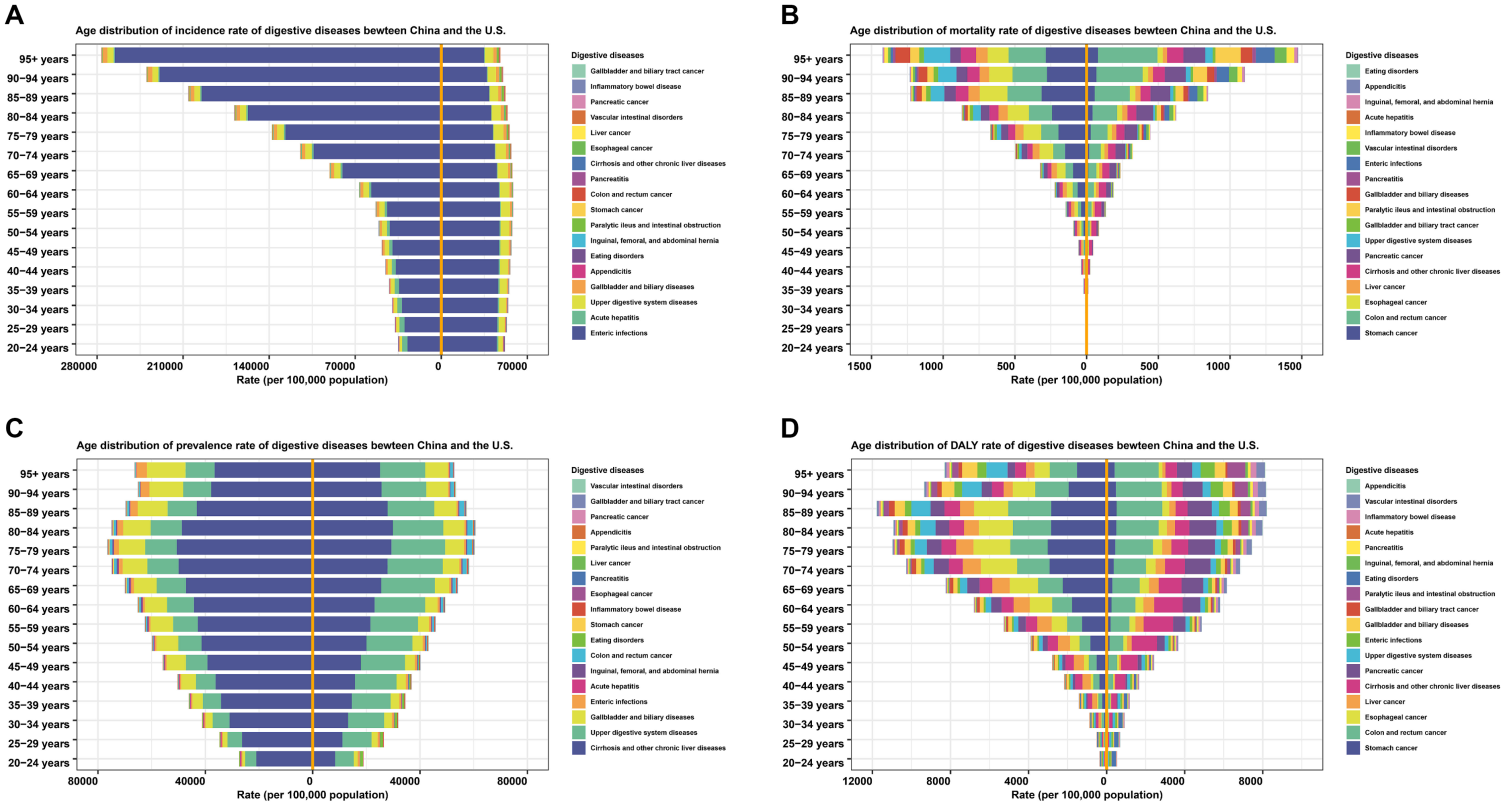
Comparison of incidence, mortality, prevalence, and DALY rates of digestive diseases between China and the United States by age in 2019. **(A)** Incidence. **(B)** Mortality. **(C)** Prevalence. **(D)** DALY. DALY, disability-adjusted life year. The legends and stacked areas were arranged from the middle line to both sides in descending order based on the age-standardized rates of digestive diseases in China in 2019.

Variations in the age range of the digestive disease burden were also evident. The overall incidence rates of 18 digestive diseases in China rose as people grew older. Simultaneously, the rates in the US showed a slight upward trajectory followed by a downward trend. The incidence rate of enteric infections, which constituted the largest portion of total rates, had a major effect on both nations. The mortality rate in both China and the US showed a consistent upward trend as age advanced. In both countries, individuals between the ages of 70 and 84 exhibited a greater prevalence rate, primarily attributed to alterations in cirrhosis and other chronic liver ailments. People aged 85–89 had the highest DALY rate for China and the US. The rates of four indicators for the majority of individual diseases demonstrated either a consistent upward trend or an initial upward trend followed by a subsequent decline, while the incidence rate of enteric infections in the US decreased with advancing age (Figure 5).

## Discussion

Based on the GBD 2019, we aimed to compare the burden of digestive diseases between China and the US, which were representatives of developing and developed countries. The changing trend of disease burden in the US can provide us with insight into the direction of China’s disease burden development. In the past thirty years, China bore a greater burden of digestive diseases compared to the US, except for the period between 2011 and 2014, when the US experienced a higher incidence rate. Although the burden of individual digestive diseases varied between the two countries, they both shared similarities in burden of certain disease and overall changing trends. The burden of digestive diseases also differed depending on age and sex.

The majority of disparities in digestive disease burden can be attributed to social and economic inequalities among nations at the population level (23). China is one of the largest developing countries in the world, accounting for approximately one-fifth of the world’s population. China and other countries with similar economic and social advancement find themselves in a vulnerable situation when public health concerns are being tackled through disease control and prevention. Health economics evaluation is essential for developing countries’ health investment and strategy optimization (24). The results of our research indicated that there were discrepancies and resemblances between the largest developing and developed nations. This not only offers an insight into the future trends of digestive disease burden in developing nations and serves as a guide for making timely adjustments to health policies, but also reveals the common problems between the two countries and advocates collaborate to discover solutions.

Firstly, the undulatory incidence rates of the two countries caught our eyes. The overwhelming proportion enteric infections occupied in the incidence rates of digestive diseases explained the critical impact of its rates on overall morbidity. Recently, increasing incidence and prevalence rates in both countries have worried people. The increasing incidence rate with age in China and its aging trends make the situation severe. Although the incidence rate of enteric infections in the US decreased with age, the mortality rate increased considerably during the past 30 years, which has not been reported before. Previous studies focused much on enteric infections in children under five years old (25, 26). Still, a study also pointed out an exceptionally high burden in adults over 70 years, explaining the high burden of *Clostridium difficile* in high-income countries might be the reason (27). In the older adults, the incidence of surgical site infections after colorectal surgery is also elevated, significantly straining healthcare resources, disease management, and economic stability (28, 29). Our finding of the continuing prevalence of enteric infections is consistent with Wang’s global study (1). Both highlight the need for health policy in enteric infections to control the disease epidemic in developing and developed countries.

The age-standardized prevalence rates of cirrhosis and other chronic liver diseases in both countries were another cause for concern. In China, viral hepatitis has been a major contributor to chronic liver diseases, cirrhosis, and liver cancer. A study conducted with GBD 2016 data revealed that hepatitis B was still the primary source of cirrhosis and other chronic liver diseases in China (30). The Chinese government’s implementation of comprehensive immunization programs (31–33), resulted in a decrease in the burden of cirrhosis and other chronic liver diseases. In developed nations, the incidence of viral hepatitis is declining as a result of advancements in disease prevention, diagnosis, and therapies (12). However, the prevalence of cirrhosis has grown in the last twenty years in the US, with incident cirrhosis diagnoses being highest and rising disproportionately among younger Americans (34, 35). As people’s quality of life improves, there will be a corresponding rise in injection drug use, alcohol misuse, and metabolic syndrome, ultimately resulting in an increase in end-stage liver diseases (36). In spite of the fact that mortality and disability rates of cirrhosis and other chronic liver diseases have decreased considerably in recent years, Chinese society is currently undergoing a major transformation in its economic, social, and cultural systems. China should capitalize on the current situation of cirrhosis in the US and then devise effective strategies to address the disease. It is imperative for the US to give sufficient consideration to the escalating cirrhosis burden and promptly develop pertinent policies to mitigate the exacerbation of this burden.

Earlier research indicated elevated mortality and DALY rates associated with esophageal cancer in males (5, 14, 37, 38), predominantly attributed to prolonged smoking and alcohol intake (39, 40). The higher ranks of mortality and DALY rates in esophageal cancer among male digestive diseases in China are partially supported by this. Alongside the extensive findings of research suggesting that the utilization of estrogen and oral contraceptives leads to a lower burden of colon and rectum cancer among females (17, 41, 42), our study also revealed that the females had higher ranks of DALY rate in colon and rectum cancer across 18 digestive disorders in both countries. The lower disease burden compared to males masks the high mortality and DALY rates of colon and rectum cancer in digestive disorders among females. Both China and the US should be aware of the significant impact of colon and rectum cancer on female digestive diseases and take appropriate action based on the prevailing burden trends. Although the disease still had more severe consequences in developed countries (43), countries with middle-to-high human development index have experienced a significant surge in both the incidence and mortality (44). A rapid increase in the burden in low SDI and middle SDI countries in Asia and Africa was also noted (42). It is essential to both reduce the growing pressure in China and keep decreasing the immense pressure in the US. A comprehensive strategy that integrates enhanced prevention, optimized diagnostic methods, improved treatment protocols, and effective health policies is crucial to reduce the pressure of colon and rectum cancer. The importance of early diagnosis cannot be overstated. Recent advances in deep learning algorithms for histopathological image analysis in colorectal cancer suggest significant potential improvements in the accuracy and efficiency of detection (45, 46).

To the best of our knowledge, this is the first study to thoroughly analyze the temporal trends of the impact of digestive illnesses in China and the US, employing GBD 2019 data classified by sex and age. Exploring the disparities in the burden of digestive diseases between the two countries can provide us with a clearer insight into the current state of the world’s two most extensive socio-economic systems, which can be beneficial for global, regional, and national health policy-making departments. Moreover, it has provided an in-depth analysis of the current situation and progression in two countries that suffer heavy burden from digestive diseases, which has a beneficial effect on the prevention and management of the diseases. The study is not without its limitations, as the data was derived from estimation and mathematical modeling (18, 19, 21), and the 95% UI of overall disease burden was obtained by directly adding 18 digestive diseases, which to some extent deviates from the accurate value.

## Conclusion

Digestive diseases pose a significant burden for both China and the US. In spite of some commonalities in the burden of digestive diseases between China and the US, there are distinct variations between the two countries. The impact of digestive diseases differs significantly between the two nations, contingent upon the particular disease category, sex, and age. Targeted interventions and urgent measures should be taken in China and the US to control the specific burden based on their different epidemic degree of digestive disease.

## Data availability statement

Publicly available datasets were analyzed in this study. This data can be found at: https://ghdx.healthdata.org/.

## Author contributions

JP: Conceptualization, Data curation, Formal analysis, Visualization, Writing – original draft. HX: Data curation, Formal analysis, Visualization, Writing – original draft. SH: Data curation, Formal analysis, Visualization, Writing – original draft. XS: Data curation, Formal analysis, Writing – review & editing. PW: Data curation, Formal analysis, Writing – original draft. QC: Data curation, Formal analysis, Writing – original draft. WZ: Writing – review & editing. LS: Writing – review & editing. YP: Writing – review & editing. FY: Conceptualization, Writing – review & editing. XT: Conceptualization, Writing – review & editing.

## Supplementary material

SUPPLEMENTARY FIGURE S1 Trends of 18 individual digestive diseases in age-standardized prevalence rates in China and the United States, 1990–2019. **(A)** Acute hepatitis. **(B)** Appendicitis. **(C)** Cirrhosis and other chronic liver diseases. **(D)** Colon and rectum cancer. **(E)** Eating disorders. **(F)** Enteric infections. **(G)** Esophageal cancer. **(H)** Gallbladder and biliary diseases. **(I)** Gallbladder and biliary tract cancer. **(J)** Inflammatory bowel disease. **(K)** Inguinal, femoral, and abdominal hernia. **(L)** Liver cancer. **(M)** Pancreatic cancer. **(N)** Pancreatitis. **(O)** Paralytic ileus and intestinal obstruction. **(P)** Stomach cancer. **(Q)** Upper digestive system diseases. **(R)** Vascular intestinal disorders.

SUPPLEMENTARY FIGURE S2 Trends of 18 individual digestive diseases in age-standardized mortality rates in China and the United States, 1990–2019. **(A)** Acute hepatitis. **(B)** Appendicitis. **(C)** Cirrhosis and other chronic liver diseases. **(D)** Colon and rectum cancer. **(E)** Eating disorders. **(F)** Enteric infections. **(G)** Esophageal cancer. **(H)** Gallbladder and biliary diseases. **(I)** Gallbladder and biliary tract cancer. **(J)** Inflammatory bowel disease. **(K)** Inguinal, femoral, and abdominal hernia. **(L)** Liver cancer. **(M)** Pancreatic cancer. **(N)** Pancreatitis. **(O)** Paralytic ileus and intestinal obstruction. **(P)** Stomach cancer. **(Q)** Upper digestive system diseases. **(R)** Vascular intestinal disorders.

SUPPLEMENTARY FIGURE S3 Trends of 18 individual digestive diseases in age-standardized DALY rates in China and the United States, 1990–2019. **(A)** Acute hepatitis. **(B)** Appendicitis. **(C)** Cirrhosis and other chronic liver diseases. **(D)** Colon and rectum cancer. **(E)** Eating disorders. **(F)** Enteric infections. **(G)** Esophageal cancer. **(H)** Gallbladder and biliary diseases. **(I)** Gallbladder and biliary tract cancer. **(J)** Inflammatory bowel disease. **(K)** Inguinal, femoral, and abdominal hernia. **(L)** Liver cancer. **(M)** Pancreatic cancer. **(N)** Pancreatitis. **(O)** Paralytic ileus and intestinal obstruction. **(P)** Stomach cancer. **(Q)** Upper digestive system diseases. **(R)** Vascular intestinal disorders. DALY, disability-adjusted life year.
