# Supplementary figures and images for: Comparison of the burden of digestive diseases between China and the United States from 1990 to 2019
Source: Front Public Health. 2024 May 17;12:1376406. doi: 10.3389/fpubh.2024.1376406 (PMC11140071; doi:10.3389/fpubh.2024.1376406)

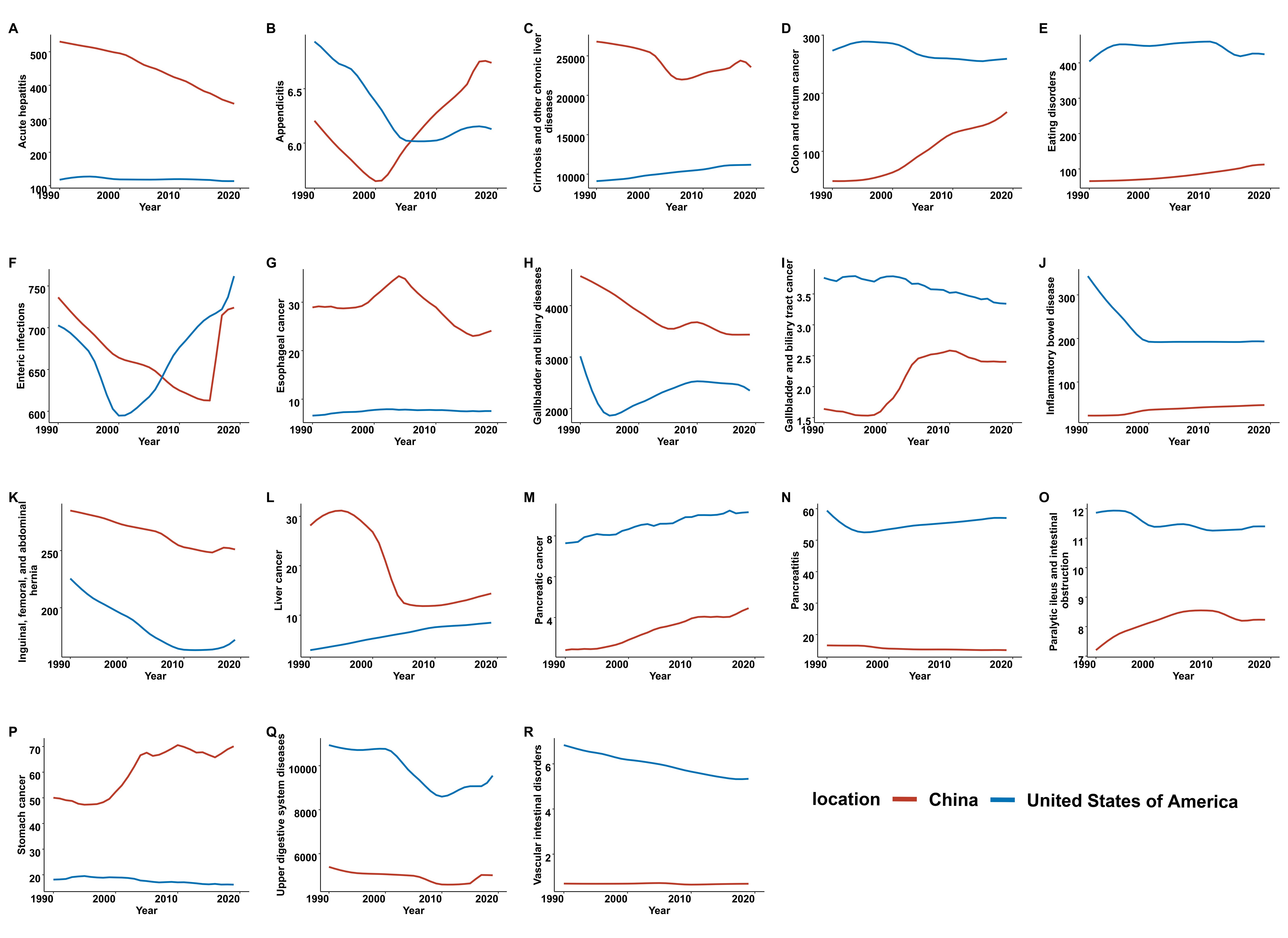

Supplement: SUPPLEMENTARY FIGURE S1 — Trends of 18 individual digestive diseases in age-standardized prevalence rates in China and the United States, 1990–2019. (A) Acute hepatitis. (B) Appendicitis. (C) Cirrhosis and other chronic liver diseases. (D) Colon and rectum cancer. (E) Eating disorders. (F) Enteric infections. (G) Esophageal cancer. (H) Gallbladder and biliary diseases. (I) Gallbladder and biliary tract cancer. (J) Inflammatory bowel disease. (K) Inguinal, femoral, and abdominal hernia. (L) Liver cancer. (M) Pancreatic cancer. (N) Pancreatitis. (O) Paralytic ileus and intestinal obstruction. (P) Stomach cancer. (Q) Upper digestive system diseases. (R) Vascular intestinal disorders. [file Image_1.JPEG]

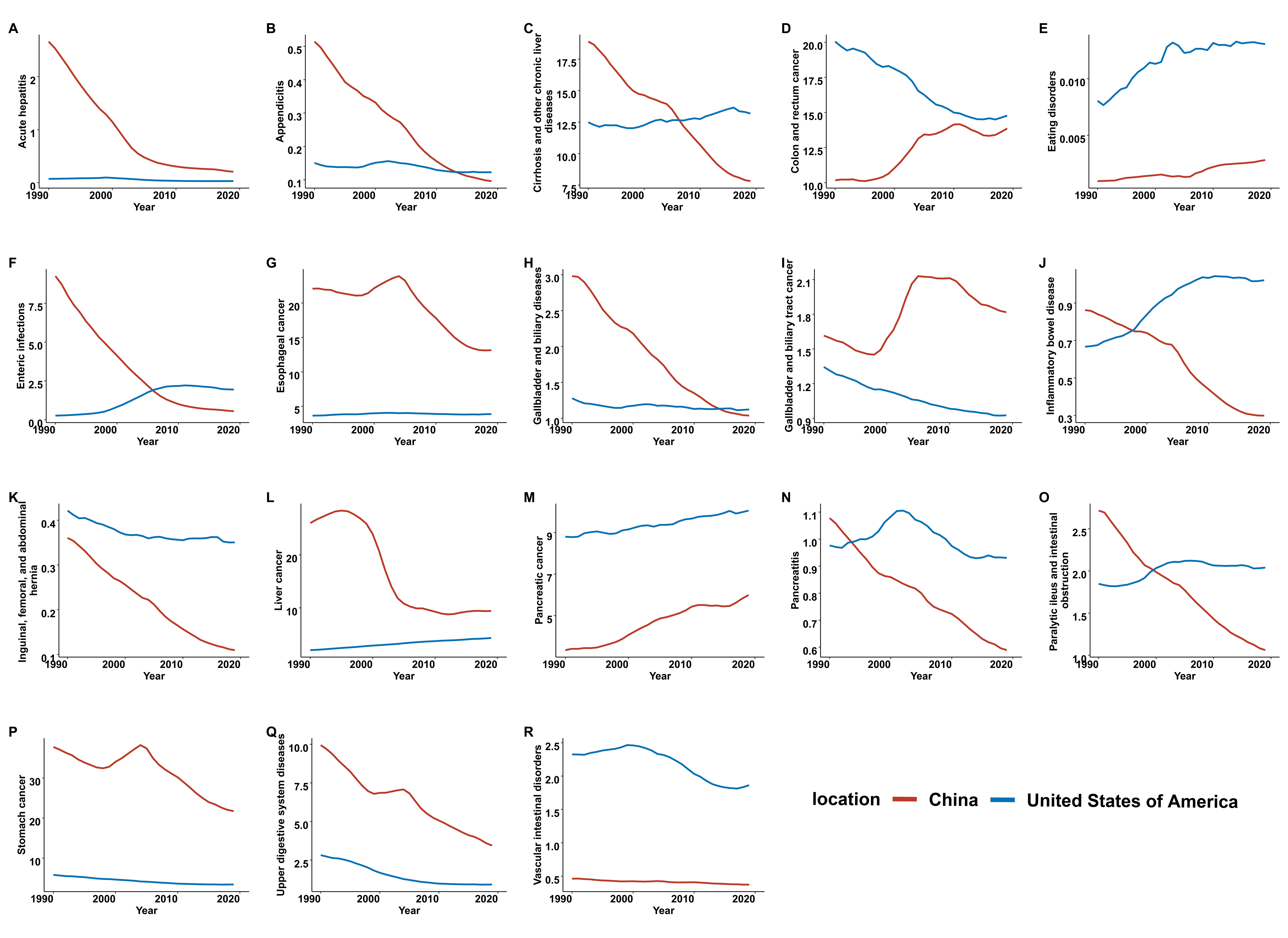

Supplement: SUPPLEMENTARY FIGURE S2 — Trends of 18 individual digestive diseases in age-standardized mortality rates in China and the United States, 1990–2019. (A) Acute hepatitis. (B) Appendicitis. (C) Cirrhosis and other chronic liver diseases. (D) Colon and rectum cancer. (E) Eating disorders. (F) Enteric infections. (G) Esophageal cancer. (H) Gallbladder and biliary diseases. (I) Gallbladder and biliary tract cancer. (J) Inflammatory bowel disease. (K) Inguinal, femoral, and abdominal hernia. (L) Liver cancer. (M) Pancreatic cancer. (N) Pancreatitis. (O) Paralytic ileus and intestinal obstruction. (P) Stomach cancer. (Q) Upper digestive system diseases. (R) Vascular intestinal disorders. [file Image_2.JPEG]

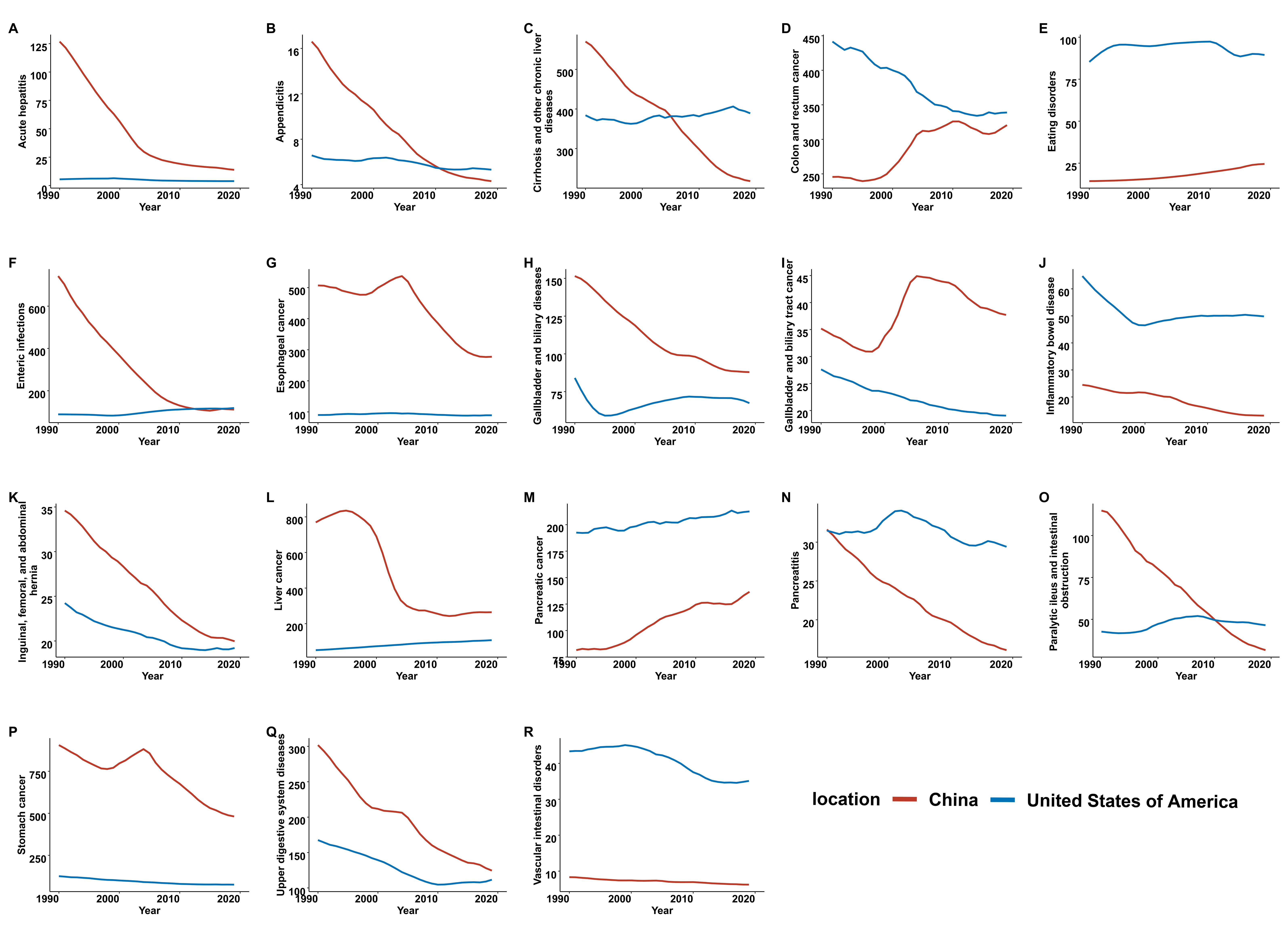

Supplement: SUPPLEMENTARY FIGURE S3 — Trends of 18 individual digestive diseases in age-standardized DALY rates in China and the United States, 1990–2019. (A) Acute hepatitis. (B) Appendicitis. (C) Cirrhosis and other chronic liver diseases. (D) Colon and rectum cancer. (E) Eating disorders. (F) Enteric infections. (G) Esophageal cancer. (H) Gallbladder and biliary diseases. (I) Gallbladder and biliary tract cancer. (J) Inflammatory bowel disease. (K) Inguinal, femoral, and abdominal hernia. (L) Liver cancer. (M) Pancreatic cancer. (N) Pancreatitis. (O) Paralytic ileus and intestinal obstruction. (P) Stomach cancer. (Q) Upper digestive system diseases. (R) Vascular intestinal disorders. DALY, disability-adjusted life year. [file Image_3.JPEG]
