# Supplementary material for: Comparison of the burden of digestive diseases between China and the United States from 1990 to 2019
Source: Front Public Health. 2024 May 17;12:1376406. doi: 10.3389/fpubh.2024.1376406 (PMC11140071; doi:10.3389/fpubh.2024.1376406)
Supplement: Supplementary file 4 [file Table_1.DOCX]

| **Supplementary Table 1** Change of Incidence, Prevalence, Deaths, and DALYs in 2019 for 18 Individual Digestive Diseases in China.  death  death_Rate  prevalence  prevalence_Rate  DALYs  DALYs_Rate | | | | | | | | |
| --- | --- | --- | --- | --- | --- | --- | --- | --- |
| **Location** | **Incidence** | | **Mortality** | | **Prevalence** | | **DALYs** | |
|  | **AAPC, 95%CI** | ***P*-Value** | **AAPC, 95%CI** | ***P*-Value** | **AAPC, 95%CI** | ***P*-Value** | **AAPC, 95%CI** | ***P*-Value** |
| Acute hepatitis | -1.30*(-1.36--1.24) | < 0.001 | -8.64*(-9.19--8.09) | < 0.001 | -1.49*(-1.53--1.45) | < 0.001 | -7.35*(-7.64--7.05) | < 0.001 |
| Appendicitis | 0.26*(0.20-0.32) | < 0.001 | -5.69*(-5.94--5.44) | < 0.001 | 0.29*(0.24-0.35) | < 0.001 | -4.58*(-4.84--4.32) | < 0.001 |
| Cirrhosis and other chronic liver diseases | -0.62*(-0.72--0.52) | < 0.001 | -3.03*(-3.14--2.91) | < 0.001 | -0.44*(-0.53--0.35) | < 0.001 | -3.28*(-3.39--3.17) | < 0.001 |
| Colon and rectum cancer | 3.11*(2.87-3.35) | < 0.001 | 1.05*(0.82-1.28) | < 0.001 | 4.38*(4.20-4.55) | < 0.001 | 0.91*(0.71-1.10) | < 0.001 |
| Eating disorders | 1.40*(1.37-1.42) | < 0.001 | 3.85*(3.36-4.34) | < 0.001 | 1.89*(1.86-1.93) | < 0.001 | 1.89*(1.85-1.93) | < 0.001 |
| Enteric infections | 0.21*(0.05-0.37) | 0.01 | -9.32*(-9.57--9.07) | < 0.001 | 0.09(-0.07-0.26) | 0.272 | -6.31*(-6.84--5.77) | < 0.001 |
| Esophageal cancer | -1.43*(-1.58--1.29) | < 0.001 | -1.83*(-1.95--1.71) | < 0.001 | -0.64*(-0.79--0.49) | < 0.001 | -2.07*(-2.20--1.95) | < 0.001 |
| Gallbladder and biliary diseases | 1.03*(0.93-1.12) | < 0.001 | -3.60*(-4.03--3.18) | < 0.001 | -0.99*(-1.01--0.96) | < 0.001 | -1.86*(-1.99--1.74) | < 0.001 |
| Gallbladder and biliary tract cancer | 0.82*(0.70-0.95) | < 0.001 | 0.41*(0.29-0.52) | < 0.001 | 1.34*(1.20-1.49) | < 0.001 | 0.26*(0.14-0.37) | < 0.001 |
| Inflammatory bowel disease | 2.50*(2.44-2.56) | < 0.001 | -3.63*(-3.85--3.41) | < 0.001 | 2.53*(2.40-2.67) | < 0.001 | -2.17*(-2.31--2.03) | < 0.001 |
| Inguinal, femoral, and abdominal hernia | 0.91*(0.83-1.00) | < 0.001 | -4.08*(-4.24--3.92) | < 0.001 | -0.44*(-0.51--0.37) | < 0.001 | -1.87*(-2.02--1.72) | < 0.001 |
| Liver cancer | -3.13*(-3.35--2.90) | < 0.001 | -3.42*(-3.72--3.12) | < 0.001 | -2.39*(-2.62--2.16) | < 0.001 | -3.60*(-3.88--3.31) | < 0.001 |
| Pancreatic cancer | 2.07*(1.81-2.33) | < 0.001 | 2.02*(1.74-2.29) | < 0.001 | 2.13*(1.89-2.37) | < 0.001 | 1.78*(1.53-2.02) | < 0.001 |
| Pancreatitis | -1.21*(-1.31--1.10) | < 0.001 | -2.09*(-2.21--1.97) | < 0.001 | -0.32*(-0.35--0.29) | < 0.001 | -2.31*(-2.47--2.15) | < 0.001 |
| Paralytic ileus and intestinal obstruction | 0.28*(0.26-0.31) | < 0.001 | -3.22*(-3.31--3.13) | < 0.001 | 0.46*(0.44-0.48) | < 0.001 | -4.37*(-4.63--4.12) | < 0.001 |
| Stomach cancer | -0.70*(-0.88--0.51) | < 0.001 | -1.90*(-2.13--1.67) | < 0.001 | 1.17*(0.74-1.60) | < 0.001 | -2.17*(-2.34--2.00) | < 0.001 |
| Upper digestive system diseases | -0.37*(-0.40--0.34) | < 0.001 | -3.62*(-3.84--3.40) | < 0.001 | -0.24*(-0.28--0.20) | < 0.001 | -2.98*(-3.17--2.78) | < 0.001 |
| Vascular intestinal disorders | -0.20*(-0.26--0.15) | < 0.001 | -0.78*(-0.94--0.61) | < 0.001 | -0.02(-0.07-0.04) | 0.547 | -1.02*(-1.14--0.89) | < 0.001 |
| **DALYs**, disability-adjust life years; **AAPC,** average annual percentage change; **95%CI,** 95% confidence intervals.  *Indicates that the AAPC is significantly different from zero at the alpha = 0.05 level | | | | | | | | |
