# Supplementary material for: Comparison of the burden of digestive diseases between China and the United States from 1990 to 2019
Source: Front Public Health. 2024 May 17;12:1376406. doi: 10.3389/fpubh.2024.1376406 (PMC11140071; doi:10.3389/fpubh.2024.1376406)
Supplement: Supplementary file 5 [file Table_2.DOCX]

| **Supplementary Table 2** Change of Incidence, Prevalence, Deaths, and DALYs in 2019 for 18 Individual Digestive Diseases in the United States.  death  death_Rate  prevalence  prevalence_Rate  DALYs  DALYs_Rate | | | | | | | | |
| --- | --- | --- | --- | --- | --- | --- | --- | --- |
| **Location** | **Incidence** | | **Mortality** | | **Prevalence** | | **DALYs** | |
|  | **AAPC, 95%CI** | ***P*-Value** | **AAPC, 95%CI** | ***P*-Value** | **AAPC, 95%CI** | ***P*-Value** | **AAPC, 95%CI** | ***P*-Value** |
| Acute hepatitis | -0.06(-0.20-0.07) | 0.354 | -3.09*(-3.43--2.75) | < 0.001 | -0.15*(-0.29-0) | 0.049 | -1.19*(-1.34--1.04) | < 0.001 |
| Appendicitis | -0.41*(-0.46--0.37) | < 0.001 | -0.71*(-0.95--0.46) | < 0.001 | -0.42*(-0.46--0.37) | < 0.001 | -0.69*(-0.93--0.44) | < 0.001 |
| Cirrhosis and other chronic liver diseases | 0.37*(0.21-0.53) | < 0.001 | 0.20*(0.02-0.39) | 0.031 | 0.72*(0.70-0.73) | < 0.001 | 0.07(-0.14-0.29) | 0.496 |
| Colon and rectum cancer | -0.49*(-0.72--0.27) | < 0.001 | -1.06*(-1.24--0.88) | < 0.001 | -0.18*(-0.24--0.12) | < 0.001 | -0.88*(-1.13--0.64) | < 0.001 |
| Eating disorders | 0.31*(0.23-0.38) | < 0.001 | 1.89*(1.62-2.15) | < 0.001 | 0.16*(0.01-0.31) | 0.037 | 0.16(0-0.32) | 0.051 |
| Enteric infections | 0.18(-0.03-0.39) | 0.091 | 7.17*(6.71-7.64) | < 0.001 | 0.26*(0.16-0.36) | < 0.001 | 0.97*(0.80-1.15) | < 0.001 |
| Esophageal cancer | 0.17*(0.11-0.23) | < 0.001 | 0.21*(0.11-0.31) | < 0.001 | 0.44*(0.32-0.55) | < 0.001 | -0.02(-0.17-0.13) | 0.768 |
| Gallbladder and biliary diseases | -0.91*(-1.24--0.59) | < 0.001 | -0.45*(-0.62--0.28) | < 0.001 | -0.82*(-0.92--0.72) | < 0.001 | -0.76*(-0.86--0.66) | < 0.001 |
| Gallbladder and biliary tract cancer | -0.85*(-1.05--0.65) | < 0.001 | -1.29*(-1.36--1.22) | < 0.001 | -0.39*(-0.68--0.09) | 0.011 | -1.27*(-1.33--1.20) | < 0.001 |
| Inflammatory bowel disease | -1.36*(-1.45--1.28) | < 0.001 | 1.50*(1.38-1.62) | < 0.001 | -1.97*(-2.06--1.87) | < 0.001 | -0.85*(-0.90--0.81) | < 0.001 |
| Inguinal, femoral, and abdominal hernia | -0.65*(-0.71--0.58) | < 0.001 | -0.63*(-0.77--0.49) | < 0.001 | -0.94*(-0.99--0.88) | < 0.001 | -0.81*(-0.89--0.72) | < 0.001 |
| Liver cancer | 2.98*(2.91-3.04) | < 0.001 | 2.64*(2.57-2.70) | < 0.001 | 3.68*(3.61-3.75) | < 0.001 | 2.57*(2.51-2.64) | < 0.001 |
| Pancreatic cancer | 0.53*(0.49-0.57) | < 0.001 | 0.48*(0.25-0.70) | < 0.001 | 0.67*(0.36-0.98) | < 0.001 | 0.37*(0.17-0.57) | < 0.001 |
| Pancreatitis | -0.68*(-0.73--0.64) | < 0.001 | -0.13(-0.31-0.06) | 0.175 | -0.14*(-0.16--0.11) | < 0.001 | -0.21*(-0.41--0.02) | 0.033 |
| Paralytic ileus and intestinal obstruction | -0.22*(-0.24--0.19) | < 0.001 | 0.34*(0.15-0.53) | < 0.001 | -0.14*(-0.18--0.10) | < 0.001 | 0.32*(0.17-0.48) | < 0.001 |
| Stomach cancer | -1.26*(-1.44--1.07) | < 0.001 | -1.83*(-1.98--1.67) | < 0.001 | -0.38*(-0.67--0.09) | 0.009 | -1.76*(-1.82--1.69) | < 0.001 |
| Upper digestive system diseases | -0.44*(-0.59--0.29) | < 0.001 | -3.83*(-4.02--3.64) | < 0.001 | -0.45*(-0.68--0.22) | < 0.001 | -1.43*(-1.59--1.26) | < 0.001 |
| Vascular intestinal disorders | -0.69*(-0.72--0.65) | < 0.001 | -0.76*(-0.89--0.63) | < 0.001 | -0.85*(-0.89--0.80) | < 0.001 | -0.72*(-0.84--0.60) | < 0.001 |
| **DALYs**, disability-adjust life years; **AAPC,** average annual percentage change; **95%CI,** 95% confidence intervals.  *Indicates that the AAPC is significantly different from zero at the alpha = 0.05 level | | | | | | | | |
